# Long-Term Survival Outcomes of NCRT With Surgery vs Surgery With Adjuvant Therapy for ESCC: A Single-Center Prospective Phase 3 Randomized Clinical Trial

**DOI:** 10.1001/jamanetworkopen.2025.50307

**Published:** 2026-01-05

**Authors:** Wenwu He, Zhiyu Li, Qin Xie, Yongtao Han, Lin Peng, Chenghao Wang, Kangning Wang, Guangyuan Liu, Haojun Li, Qiang Zhou, Jialong Li, Huan Zhang, Wenguang Xiao, Qiang Fang, Yan Miao, Xuefeng Leng

**Affiliations:** 1 Department of Thoracic Surgery, Sichuan Clinical Research Center for Cancer, Sichuan Cancer Hospital & Institute, Sichuan Cancer Center, University of Electronic Science and Technology of China, Chengdu, China; 2 Department of Thoracic Surgery, The People’s Hospital of Leshan, Leshan, China

## Abstract

**Importance:**

The optimal treatment strategy for locally advanced esophageal squamous cell carcinoma (ESCC) remains controversial. Neoadjuvant chemoradiotherapy (NCRT) followed by surgery and surgery with adjuvant therapy (AT) are commonly used approaches.

**Objectives:**

To compare the long-term survival outcomes, safety, and pathological benefits of NCRT followed by surgery vs surgery with AT in patients with locally advanced ESCC, and to evaluate the survival impact of pathological complete response (pCR) in the NCRT subgroup.

**Design, Setting, and Participants:**

This prospective, randomized, open-label phase 3 trial was conducted at Sichuan Cancer Hospital, China, between January 2018 and April 2020. Eligible patients were aged 18 to 75 years; had histologically confirmed, resectable, locally advanced thoracic ESCC, staged as cT1N+M0 or cT2-4aNxM0; were expected to survive more than 6 months; and had adequate organ function. Key exclusion included prior malignant neoplasms, distant or cervical lymph node metastasis, contraindications to surgery, and prior gastrectomy precluding reconstruction. Data were analyzed April to December 2024.

**Interventions:**

Patients in the NCRT group received intensity-modulated radiotherapy (40 Gy in 20 fractions) with concurrent paclitaxel and carboplatin followed by surgery. Patients in the AT group received an adjuvant chemoradiotherapy regimen designed by a multidisciplinary team.

**Main Outcomes and Measures:**

The primary end point was overall survival (OS). Secondary end points included disease-free survival (DFS), pathological outcomes, treatment-related toxic effects, and perioperative complications.

**Results:**

A total of 254 patients were initially enrolled; after exclusions and randomization, 118 patients in the NCRT group (median [IQR] age, 62 [54-66] years; 102 [86.4%] male) and 112 patients in the AT group (median [IQR] age, 63 [55-66] years; 97 [86.6%] male) were included in the analysis. After a median (IQR) follow-up of 59.1 (54.4-65.9) months, there were no significant differences in OS or DFS between the groups. The 5-year OS rates were 59.2% (95% CI, 51.0%-68.8%) for the NCRT group and 59.6% (95% CI, 51.2%-69.5%) for the AT group (hazard ratio [HR], 1.01; 95% CI, 0.67-1.51; *P* = .97). The 5-year DFS rates were 53.1% (95% CI, 44.7%-63.1%) and 56.5% (95% CI, 47.9%-66.7%), respectively (HR, 1.13; 95% CI, 0.77-1.68; *P* = .53). Subgroup analysis showed that patients achieving pCR in the NCRT group had significantly improved survival outcomes, with a 5-year OS rate of 76.5% (95% CI, 63.5%-92.1%) compared with 52.1% (95% CI, 42.4%-64.1%) for non-pCR patients (HR, 0.39; 95% CI, 0.18-0.82; *P* = .01).

**Conclusions and Relevance:**

In this randomized clinical trial, there were no differences in OS or DFS for patients treated with NCRT followed by surgery or surgery with AT. However, for the patients who achieved pCR with NCRT, there were significant advantages, with markedly improved long-term outcomes. Nevertheless, neoadjuvant chemoradiotherapy did not benefit all patients; for those less likely to respond, the combination of surgery with postoperative AT remains a reasonable strategy. These findings show that it may not be appropriate to universally recommend neoadjuvant chemoradiotherapy for all patients.

**Trial Registration:**

ClinicalTrials.gov Identifier: NCT06775652

## Key Points

**Question:**

Among patients with locally advanced esophageal squamous cell carcinoma (ESCC), does neoadjuvant chemoradiotherapy (NCRT) followed by surgery improve long-term survival compared with surgery followed by adjuvant therapy (AT)?

**Findings:**

In this randomized clinical trial of 254 patients with locally advanced ESCC, the 5-year overall survival (OS) was 59.2% in the NCRT group and 59.6% in the AT group, showing no significant difference between the 2 groups.

**Meaning:**

These findings indicate that surgery followed by adjuvant therapy remains a reasonable treatment option for resectable disease, emphasizing the need to identify patients most likely to benefit from NCRT.

## Teaser

This randomized clinical trial examines whether there is a difference in overall and disease-free survival among adult patients in China with locally advanced esophageal squamous cell carcinoma (ESCC) treated with neoadjuvant chemoradiotherapy (NCRT) followed by surgery and those treated with surgery followed by adjuvant therapy,

## Introduction

Esophageal cancer (EC) is a globally significant gastrointestinal malignant neoplasm. According to GLOBOCAN 2022, EC ranks as the 11th most common cancer worldwide and the 6th leading cause of cancer-related death.^1^ In China, more than 90% of EC cases are esophageal squamous cell carcinoma (ESCC), which poses substantial treatment challenges due to its late-stage diagnosis and high recurrence rates.^2^ Despite advancements in surgical techniques, survival remains poor for patients with locally advanced ESCC treated with surgery alone, with 5-year survival rates of approximately 25%.^3^

Recently, neoadjuvant chemoradiotherapy (NCRT) combined with surgery has shown promising survival benefits. Landmark trials like CROSS^4^ have found that NCRT improves survival compared with surgery alone for locally advanced EC. Similarly, the NEOCRTEC_50_10 trial^5^ confirmed the effectiveness of NCRT in Chinese patients with ESCC, showing that NCRT significantly improves survival over surgery alone. Consequently, NCRT combined with surgery has been increasingly recommended in treatment guidelines.

Historically, surgery combined with adjuvant therapy (AT) has been an important component of treatment for EC. The Japanese JCOG9204 trial^6^ found that postoperative chemotherapy significantly improved disease-free survival (DFS) in patients with positive lymph nodes.^6^ A study by Wong et al^7^ also found that postoperative adjuvant chemoradiotherapy improves overall survival (OS) in patients with node-positive disease. Although evidence increasingly supports the superiority of NCRT combined with surgery, there remains significant clinical debate regarding the relative efficacy of NCRT vs surgery with AT. Limited studies^8,9^ have directly compared these 2 treatment strategies, and there is a notable lack of prospective research evaluating both short-term and long-term survival outcomes.

Therefore, we conducted this prospective, randomized clinical trial to clarify the long-term survival outcomes of NCRT followed by surgery vs surgery with AT in patients with locally advanced ESCC. This study aims to provide robust evidence to guide treatment optimization for locally advanced ESCC.

## Methods

### Study Design and Participants

This prospective, single-center, open-label, randomized phase 3 clinical trial was conducted at Sichuan Cancer Hospital (China) between January 2018 and April 2020. Eligible patients had histologically confirmed, resectable, locally advanced thoracic ESCC, staged as cT1N+M0 or cT2-4aNxM0 according to the American Joint Commission on Cancer eighth edition.^10^ Additional inclusion criteria were age between 18 and 75 years, expected survival of more than 6 months, and adequate organ function. Key exclusion included prior malignant neoplasm, distant or cervical lymph node metastasis, contraindications to surgery, prior gastrectomy precluding reconstruction, and inability to provide informed consent.

This protocol (Supplement 1) was approved by the institutional ethics committee, and written informed consent was obtained from all patients. This trial was conducted in accordance with the Declaration of Helsinki^11^ and the Good Clinical Practice (GCP) guidelines.^12^ This report was prepared in accordance with the Consolidated Standards of Reporting Trials (CONSORT) 2010 statement and checklist.

### Randomization and Pretreatment Evaluation

Patients were randomized (1:1) to receive either neoadjuvant chemoradiotherapy followed by surgery (NCRT group) or surgery followed by AT (AT group) using a stratified permuted block design with random numbers generated via the REDCap randomization module. Pretreatment workup included physical examination; endoscopic ultrasonography; computed tomography (CT) of the neck, chest, and abdomen; cervical ultrasonography; electrocardiogram; echocardiography; and pulmonary function tests. Bronchoscopy was performed for upper thoracic tumors, and positron emission tomography–CT was optional. Clinical staging followed the UICC TNM eighth edition.^10^

### Treatment Protocol and Assessments

In the NCRT group, patients received 2 cycles of paclitaxel (135 mg/m^2^) and carboplatin (area under the curve, 2-5) every 3 weeks, with concurrent intensity-modulated radiotherapy (IMRT; 40 Gy over 20 fractions; gross tumor volume, 44 Gy). Radiotherapy plans were reviewed before treatment. Tumor response was assessed 4 to 6 weeks after NCRT using Response Evaluation Criteria in Solid Tumors (RECIST) version 1.1 criteria.

In the AT group, surgery was performed after randomization, followed by AT based on pathological staging, as recommended by a multidisciplinary team (MDT), starting 1 month after surgery with chemoradiotherapy. Adjuvant chemotherapy used the same regimen as NCRT.

### Surgical Procedure

McKeown or Ivor Lewis esophagectomy with systematic lymphadenectomy was performed in all patients. The preferred reconstruction was a tubular gastric conduit with cervical or intrathoracic anastomosis. R0 resection was defined as negative margin. Further therapy for R1 and R2 resections was determined by an MDT.

### Assessments and Follow-up

Adverse events (AEs) were graded per the Common Terminology Criteria for Adverse Events (CTCAE) version 4.0,^13^ and postoperative complications within 30 days were classified by the Clavien-Dindo system.^14^ Follow-up was conducted every 3 months for 2 years and every 6 months for 3 to 5 years.

### Pathologic Evaluation

All surgical specimens were reviewed by experienced pathologists. Reports included tumor type, margins, tumor regression grade (Mandard score), TNM stage, vascular invasion, neural invasion, and lymph node status. Pathologic complete response (pCR) was defined as no viable tumor cells in both the primary site and resected nodes.^15^

### End Points

The primary end point was OS, defined as the time from randomization to death or last follow-up. Secondary end points included DFS (time from R0 resection to recurrence or death), safety, perioperative morbidity and mortality, R0 resection rate, and pathologic response.

### Sample Size Calculation

As one of the participating centers in the NEOCRTEC_50_10 trial, we conducted a sample size calculation based on internal data and the JCOG9907 trial. We assumed a 5-year OS rate of 60% for patients assigned to the NCRT group and 43% for those assigned to the AT group. With a 1-sided type I error of .05 and a power of 80%, using a randomization ratio of 1:1 between the experimental and control groups, and accounting for a 5% dropout rate, the intended number of randomly assigned patients was 206 (103 per group). The sample size calculation assumed an exponential distribution for survival times.

### Statistical Analysis

The analyses followed the intention-to-treat (ITT) principle. All randomized patients were analyzed in the groups to which they were originally assigned, regardless of whether they subsequently received, discontinued, or deviated from the assigned treatment. OS and DFS were calculated using the Kaplan-Meier method and compared using the log-rank test. Categorical variables between groups were compared using the χ^2^ test or Fisher exact test, while continuous variables were assessed using *t* tests or analysis of variance. A *P* value less than .05 was considered statistically significant. The Cox proportional hazards model was employed to calculate hazard ratios (HRs) and their 95% CIs. Statistical analyses were performed using SPSS version 26.0 (IBM Corp) and R version 4.2.3 (R Project for Statistical Computing).

## Results

### Treatment Compliance and Patient Characteristics

**Figure 1.**
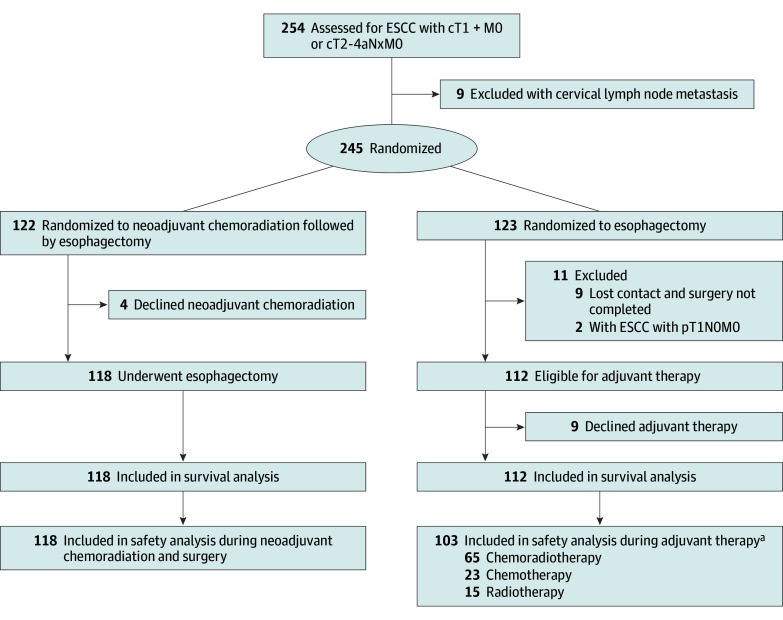
Study Flow Diagram ESCC indicates esophageal squamous cell carcinoma. ^a^Due to health issues, 11 patients switched to oral tegafur, 6 interrupted chemotherapy, and 3 interrupted radiotherapy.

Between January 2018 and April 2020, a total of 254 patients with cT1N+M0 or T2-4aNxM0 ESCC were assessed for eligibility, of whom 9 were excluded due to cervical lymph node metastasis. The remaining 245 patients were enrolled and randomly assigned to either the NCRT group (n = 122) or the AT group (n = 123). In the NCRT group, 4 patients withdrew from the study because they refused neoadjuvant therapy. Ultimately, 118 patients (median [IQR] age, 62 [54-66] years; 102 [86.4%] male) were included in the final analysis for this group. In the AT group, 11 patients were excluded, including 2 with pathological stage pT1N0M0 and 9 who were lost to follow-up and did not undergo the planned surgery, leaving 112 patients (median [IQR] age, 63 [55-66] years; 97 [86.6%] male) in this group (Figure 1). Additionally, 9 patients declined AT. Due to adverse effects or patient preference, 23 patients chose adjuvant chemotherapy (1 discontinued chemotherapy during treatment), 15 chose adjuvant radiotherapy, and 65 chose adjuvant chemoradiotherapy per protocol. Subsequently, for health-related reasons during treatment, among those who initially chose chemoradiotherapy, 11 switched to oral tegafur, 5 discontinued chemotherapy, and 3 discontinued radiotherapy. Ultimately, 46 patients completed adjuvant chemoradiotherapy as planned, for a completion rate of 46 of 112 (41.1%). Most primary tumors were located in the middle or lower third of the esophagus (209 of 230 patients [90.9%]). Among the enrolled patients, 65 (28.3%) were clinical stage II, 137 (59.6%) were clinical stage III, and 22 (9.6%) were clinical stage IVA. The baseline clinical characteristics of enrolled patients were well balanced (eTable 1 in Supplement 2).

### Safety Profile

This study compared the treatment-related AEs between the NCRT and AT groups, revealing similar overall AE patterns, with differences observed in certain specific AEs (eTable 2 in Supplement 2). In terms of hematologic toxic effects, the incidence of leukopenia and neutropenia was slightly higher in the NCRT group, but the differences were not statistically significant (any-grade leukopenia: 86.4% vs 78.6%; *P* = .17; any-grade neutropenia: 68.6% vs 56.3%; *P* = .14). For grade 3 to 4 hematologic toxic effects, leukopenia (35.6% vs 26.2%; *P* = .17) and neutropenia (32.2% vs 23.3%; *P* = .14) were more frequent in the NCRT group, but these differences did not reach statistical significance.

Regarding gastrointestinal toxic effects, the NCRT group had a significantly higher incidence of anorexia (53.4% vs 34.0%; *P* = .01) and vomiting (22.0% vs 9.7%; *P* = .04) compared with the AT group. Notably, grade 3 to 4 anorexia was also more common in the NCRT group (3.4% vs 1.0%; *P* = .01). Additionally, fatigue was more frequently observed in the NCRT group than in the AT group, but the difference was not statistically significant (50.8% vs 35.0%; *P* = .05). For radiation-related toxic effects, radiation esophageal symptoms occurred in 39.0% of patients in the NCRT group, compared with 33.7% in the AT group (*P* = .38).

### Surgical Outcomes

In the NCRT group, 2 patients had positive surgical margins postoperatively, while in the AT group, 4 patients had positive surgical margins, resulting in R0 resection rates of 98.3% vs 96.4% (*P* = .63) (eTable 3 in Supplement 2). All patients who underwent surgical treatment were included in the OS analysis. Only patients achieving R0 resection were included in the DFS analysis. McKeown esophagectomy was the predominant surgical approach in both groups, performed in 110 patients (93.2%) in the NCRT group and 101 (90.2%) in the AT group. The proportions of minimally invasive surgeries were similar between the 2 groups, at 89.8% in the NCRT group (106 patients) and 89.3% in the AT group (100 patients), with no significant differences observed. The median (IQR) numbers of lymph nodes dissected were 16 (11-22) in the NCRT group and 20 (13-29) in the AT group (*P* < .001).

The overall postoperative complication rates were 38.1% (45 of 118) in the NCRT group and 39.3% (44 of 112) in the AT group, with no significant differences between the groups (eTable 4 in Supplement 2). Anastomotic leakage occurred in 11.0% of patients (13 of 118) in the NCRT group and 14.3% (16 of 112) in the AT group, with no statistically significant difference (*P* = .58). One patient in each group died within 30 days postoperatively due to anastomotic leakage, with no significant difference between the groups.

### Pathologic Outcome

Regarding the distribution of pathologic staging, despite balanced clinical staging between the 2 groups at enrollment, 64 of 118 patients (54.2%) in the NCRT group were classified as pathologic stage I postoperatively, whereas 66 of 112 patients (58.9%) in the AT group were classified as pathologic stage III. The prevalence of positive lymph nodes was 28.8% (34 of 118) in the NCRT group vs 72.3% (81 of 112) in the AT group (*P* < .001). Moreover, there were significant differences in vascular cancer thrombus presence between the postoperative specimens of the NCRT and AT groups (10 of 118 [8.5%] vs 48 of 108 [42.9%]; *P* < .001). Similarly, significant differences were observed in neural invasion between the groups (22 of 118 [18.6%] vs 44 of 112 [39.3%]; *P* = .001). Among the 118 patients in the NCRT group, 34 (28.8%) achieved pCR after NCRT treatment (eTable 3 in Supplement 1).

### Survival Outcomes

**Figure 2.**
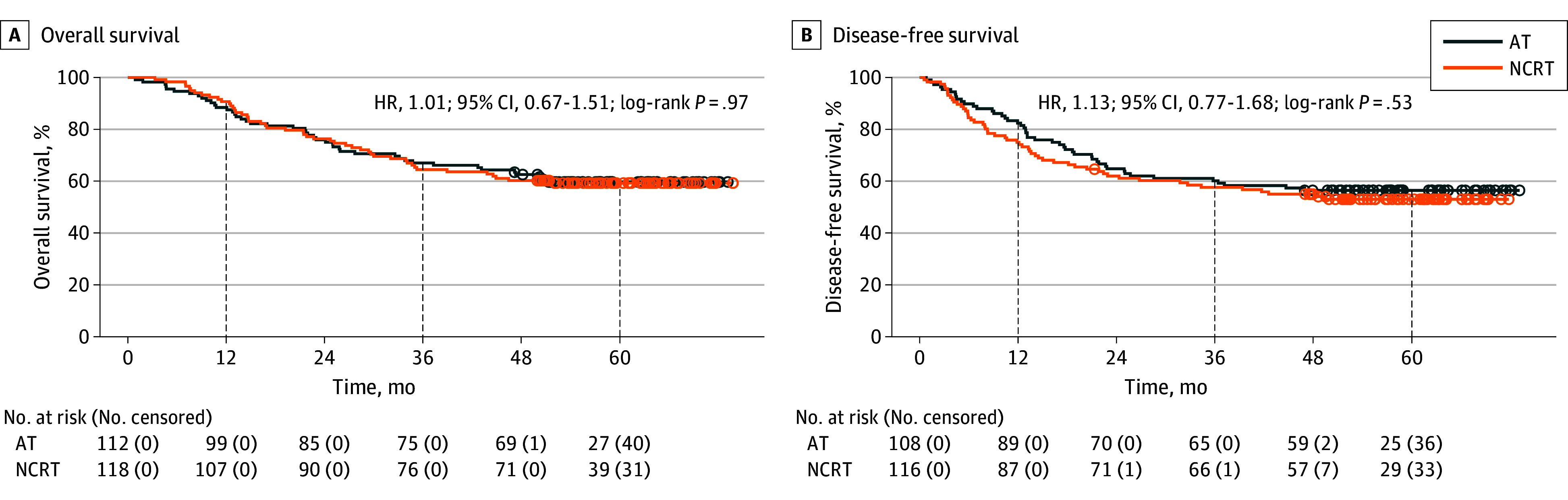
Overall Survival and Disease-Free Survival Among Neoadjuvant Chemoradiotherapy (NCRT) and Adjuvant Therapy (AT) Groups HR indicates hazard ratio.

The median (IQR) follow-up duration for survivors was 59.1 (54.4-65.9) months. Kaplan-Meier analysis revealed no significant differences in OS (HR, 1.01; 95% CI, 0.67-1.51; *P* = .97) (Figure 2A) or DFS (HR, 1.13; 95% CI, 0.77-1.68; *P* = .53) (Figure 2B) between the NCRT and AT groups. The 1-year OS rates were 90.7% (95% CI, 85.8%-96.1%) in the NCRT group and 88.4% (95% CI, 82.7%-94.5%) in the AT group. The 3-year OS rates were 64.4% (95% CI, 56.3%-73.7%) and 67.0% (95% CI, 58.8%-76.3%), respectively, and the 5-year OS rates were 59.2% (95% CI, 51.0%-68.8%) and 59.6% (95% CI, 51.2%-69.5%), respectively. The 1-year DFS rates were 75.0% (95% CI, 67.5%-83.3%) in the NCRT group and 82.4% (95% CI, 75.5%-89.9%) in the AT group. The 3-year DFS rates were 57.7% (95% CI, 49.3%-67.4%) and 60.2% (95% CI, 51.6%-70.2%), respectively, while the 5-year DFS rates were 53.1% (95% CI, 44.7%-63.1%) and 56.5% (95% CI, 47.9%-66.7%), respectively.

**Figure 3.**
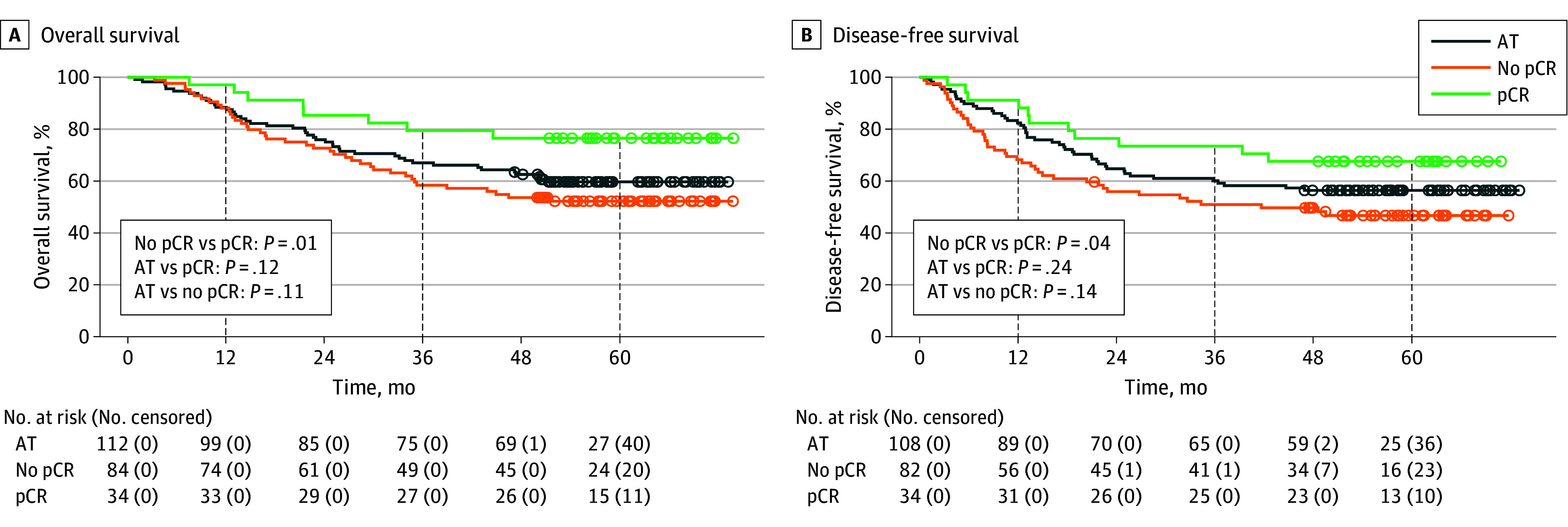
Overall and Disease-Free Survival in Neoadjuvant Chemoradiotherapy (NCRT) Subgroups (Pathologic Complete Response [pCR] and no pCR) Compared With the Adjuvant Therapy (AT) Group

**Figure 4.**
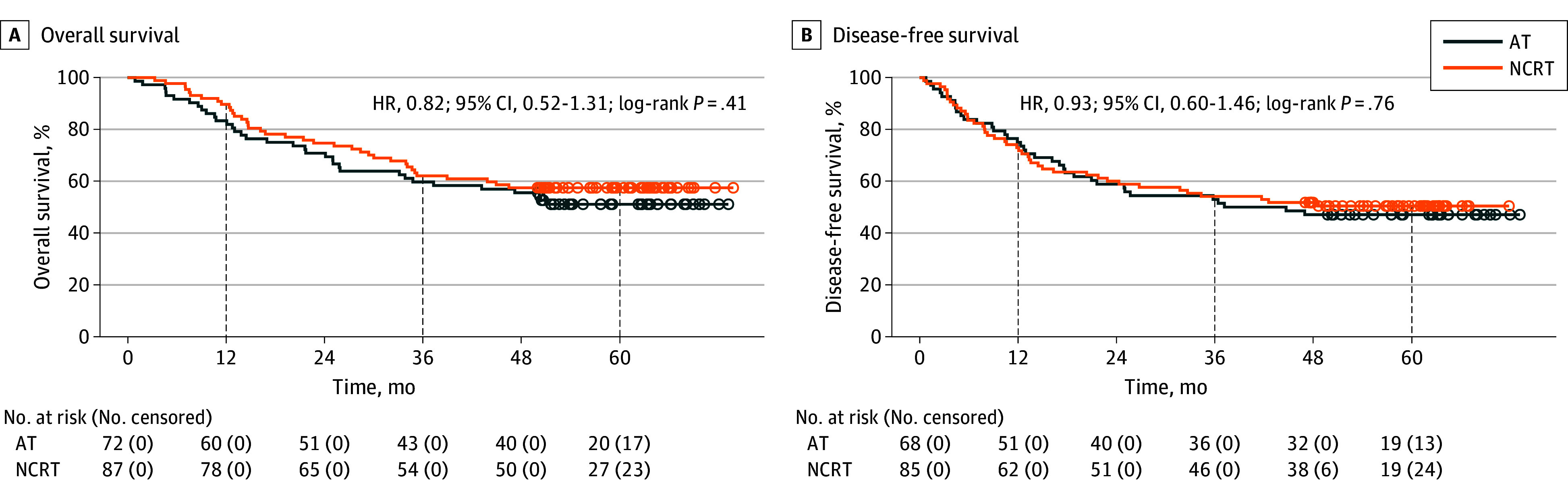
Overall and Disease-Free Survival Among Patients in the Neoadjuvant Chemoradiotherapy (NCRT) and Adjuvant Therapy (AT) Groups With cTNM III-IVA Disease HR indicates hazard ratio.

In the NCRT subgroup analysis, significant differences were observed in OS (HR, 0.39; 95% CI, 0.18-0.82; *P* = .01) (Figure 3A) and DFS (HR, 0.50; 95% CI, 0.26-0.97; *P* = .04) (Figure 3B) between the pCR and no-pCR groups. The 1-year OS rates were 97.1% (95% CI, 91.5%-100%) in the pCR group and 88.1% (95% CI, 81.4%-95.3%) in the no-pCR group. The 3-year OS rates were 79.4% (95% CI, 66.9%-94.2%) and 58.3% (95% CI, 48.7%-69.9%), respectively, and the 5-year OS rates were 76.5% (95% CI, 63.5%-92.1%) and 52.1% (95% CI, 42.4%-64.1%), respectively. The 1-year DFS rates were 91.2% (95% CI, 82.1%-100%) in the pCR group and 68.3% (95% CI, 58.9%-79.1%) in the no-pCR group. The 3-year DFS rates were 73.5% (95% CI, 60.1%-90.0%) and 51.0% (95% CI, 41.3%-63.1%), respectively, while the 5-year DFS rates were 67.6% (95% CI, 53.6%-85.3%) and 46.8% (95% CI, 37.0%-59.2%), respectively. In the patients with cTNM stage III to IVA disease, no significant differences were observed in OS (HR, 0.82; 95% CI, 0.52-1.31; *P* = .41) (Figure 4A) and DFS (HR, 0.93; 95% CI, 0.60-1.46; *P* = .76) (Figure 4B) between the NCRT and AT groups.

## Discussion

The primary finding of this study is that there were no significant differences in long-term survival outcomes, including OS and DFS, between the NCRT and AT groups. The 2 groups also showed similar R0 resection rates; however, the NCRT group demonstrated distinct advantages in terms of pathological downstaging and pCR. In the NCRT subgroup analysis, patients who achieved pCR had significantly improved long-term survival compared with those who did not, highlighting the potential benefits of NCRT in enhancing pathological responses and survival outcomes in locally advanced ESCC. For patients who fail to achieve a significant pathological response to NCRT, intensified systemic treatment and enhanced follow-up monitoring are essential.

This finding aligns with certain aspects of previous studies while also demonstrating some differences. For example, the JCOG9907 trial^8^ demonstrated that neoadjuvant chemotherapy (cisplatin and 5-fluorouracil) significantly improved OS compared with postoperative adjuvant chemotherapy. However, subgroup analysis revealed no significant survival benefit in patients with clinical stage III disease, suggesting that the efficacy of treatment strategies may depend on tumor stage and pathological response. Our study also suggests that there are no significant survival differences between the NCRT and AT groups in patients with clinical stage III disease. Similarly, the JCOG8201 study^16^ found that among patients with EC, postoperative adjuvant radiotherapy alone yielded better OS than neoadjuvant radiotherapy combined with postoperative adjuvant radiotherapy. These findings suggest that treatment benefits may vary based on both therapeutic approaches and patient characteristics. This study underscores the pathological advantages of NCRT, particularly in achieving pathological downstaging and complete response, which have also been supported by the CROSS trial^4^ and the NEOCRTEC_50_10 study.^5^ These studies demonstrated the effectiveness of NCRT in improving pathological responses and survival outcomes in EC. However, for patients who do not derive significant benefits from NCRT, future research should focus on optimizing treatment strategies that integrate the strengths of both NCRT and AT, thereby maximizing the potential for personalized treatment approaches.

The pathological advantages of NCRT, including pathological downstaging and complete response, play a pivotal role in its clinical efficacy. Achieving pCR is strongly associated with improved survival outcomes, as evidenced in this study and other trials. Patients with pCR demonstrated significantly better OS and DFS compared with those without pCR, highlighting the survival benefits conferred by robust pathological response. The mechanisms underlying these benefits may include the eradication of micrometastases, reduction in tumor burden, and suppression of aggressive tumor biology, thereby decreasing the risk of postoperative recurrence.^17,18^

Pathological downstaging further complements the benefits of pCR by reducing residual disease and potentially enhancing the efficacy of subsequent surgical resection. Several studies, including the CROSS trial^4^ and the NEOCRTEC_50_10 study,^5^ have shown that achieving pCR after NCRT correlates with reduced recurrence rates and improved survival in locally advanced ESCC. The CROSS trial^4^ reported a pCR rate of 49% for patients with ESCC, while the NEOCRTEC_50_10 trial^5^ demonstrated a pCR rate of 43.2%, with both studies showing significant survival benefits for patients with pCR. These findings underscore the clinical importance of NCRT as an integral component of treatment for locally advanced ESCC. By enhancing pCR rates and achieving effective tumor downstaging, NCRT has the potential to optimize therapeutic outcomes. Future research should focus on refining NCRT regimens to further increase pCR rates and improve the long-term prognosis of patients with ESCC.

Systemic AT is regarded as a complement to surgical resection, particularly for patients at high risk of recurrence. In our study, the OS and DFS rates in the AT group were comparable with those in the NCRT group, which may be explained by 2 factors. First, the major survival benefit of neoadjuvant chemoradiotherapy is largely confined to patients who achieve pCR; patients with poor pathological response continue to have unfavorable outcomes, and when they constitute the majority they can dilute any population-level advantage of NCRT. Second, the potential value of postoperative adjuvant treatment for patients at high risk of recurrence should not be dismissed: although high-level evidence is limited, some studies report improved long-term survival with adjuvant chemotherapy and/or radiotherapy.^6,19,20^ A plausible unifying hypothesis is that effective control of micrometastatic disease in patients with high risk—whether achieved preoperatively or postoperatively—is the key determinant of outcome. The clinical challenge going forward is to prospectively identify patients likely to attain pCR: those expected to respond well could preferentially receive neoadjuvant therapy (and thereby avoid overtreatment after surgery). For patients expected to have a poor pathological response, proceeding with upfront surgery followed by perioperative AT is also a reasonable strategy. Emphasis should be placed on optimizing systemic treatment intensity and postoperative surveillance for high-risk patients.

The application of immune-checkpoint inhibitors in both the neoadjuvant and adjuvant settings is evolving rapidly. The CheckMate-577 trial^21^ established adjuvant nivolumab as the standard of care for patients who do not achieve pCR after NCRT, and several studies^22,23^ of preoperative chemotherapy combined with immune checkpoint inhibitors have reported survival outcomes that may surpass those of conventional NCRT.^22,23^ Notably, both the CROSS^4^ and NEOCRTEC_50_10^5^ trials showed that a substantial proportion of patients who achieved pCR still developed postoperative distant metastases. Likewise, the randomized CMISG-1701^24^ trial demonstrated that although NCRT yields higher pCR rates and greater pathological downstaging than neoadjuvant chemotherapy, this did not translate into a long-term survival benefit. While optimizing neoadjuvant regimens to increase pCR rates is an important strategy to improve outcomes in locally advanced EC, the role of effective postoperative systemic therapy should not be overlooked. Future research should prioritize interventions that achieve durable, systemic control of micrometastatic disease to further improve long-term prognosis.

This study found that the overall rates of treatment-related toxic effects and postoperative complications were comparable between the NCRT and AT groups, although hematologic and gastrointestinal toxic effects were slightly higher in the NCRT group. Leukopenia and neutropenia were more frequent in the NCRT group, but the differences were not statistically significant. Additionally, gastrointestinal toxic effects, such as anorexia and vomiting, were more common in the NCRT group, though most adverse events were mild to moderate, and the incidence of severe events remained low. Postoperative complications, including anastomotic leakage and 30-day mortality rates, were similar between the 2 groups, indicating that NCRT does not significantly increase perioperative risks. Compared with the CROSS^4^ and NEOCRTEC_50_10^5^ trials, the safety profile in this study was consistent, further supporting the tolerability of NCRT. Additionally, the rate of hematologic toxic effects observed in this study were lower than that reported in the JCOG9907 trial,^8^ which involved neoadjuvant chemotherapy. This difference may reflect variations in treatment regimens and the mechanisms of toxic effects.

### Strengths and Limitations

The primary strengths of this study include its prospective randomized design, which minimizes selection bias, and a median follow-up of 59.1 months, providing robust long-term survival data. However, some limitations should be noted.

As a single-center study, the generalizability of the findings may be limited, and the relatively small sample size could restrict the statistical power for secondary end points. Future multicenter studies with larger sample sizes are needed to validate these findings and optimize treatment strategies for locally advanced ESCC.

## Conclusions

This randomized clinical trial demonstrated that NCRT followed by surgery and surgery with AT yielded comparable long-term survival outcomes in patients with locally advanced ESCC. The safety profile was comparable between the 2 groups, with no significant differences in postoperative complications or perioperative mortality. Whether given before or after surgery, the therapeutic goal is complete local tumor removal and effective control of micrometastatic disease. NCRT did not benefit all patients; for those unlikely to respond, surgery followed by AT remains a valid option. Therefore, universal endorsement of NCRT is not necessarily appropriate.

## Notes

Supplement 1.
Trial Protocol

Supplement 2. **eTable 1.** Patient Characteristics **eTable 2.** Adverse Events Between NCRT and AT Groups **eTable 3.** Surgical and Pathological Findings **eTable 4.** Postoperative Complications

Supplement 3.
Data Sharing Statement
