# Supplementary material for: Long-Term Survival Outcomes of NCRT With Surgery vs Surgery With Adjuvant Therapy for ESCC: A Single-Center Prospective Phase 3 Randomized Clinical Trial
Source: JAMA Netw Open. 2026 Jan 5;9(1):e2550307. doi: 10.1001/jamanetworkopen.2025.50307 (PMC12771256; doi:10.1001/jamanetworkopen.2025.50307)
Supplement: Supplement 1. — Trial Protocol [file jamanetwopen-e2550307-s001.pdf]

Neoadjuvant Chemoradiotherapy Combined with Surgery vs. Surgery with Adjuvant Therapy for Esophageal Squamous Cell Carcinoma: A Prospective, Randomized Phase III Clinical Trial

**Institution**	Sichuan Clinical Research Center for Cancer, Sichuan Cancer Hospital & Institute, Sichuan Cancer Center, University of Electronic Science and Technology of China
**Principal Investigator**	Yongtao Han, Wenwu He, Xuefeng Leng.
**Version Number**	V1.0
**Version Date**	September 18th, 2017

Contents

Protocol summary 3
1. Study Background 6
2. Study Objectives 7
3. Study design 8
4. Study Population 9
    4.1 Participants 9
    4.2 Inclusion criteria 9
    4.3 Exclusion criteria 10
    4.4 Removal criteria 10
    4.5 Drop-out/Withdrawal criteria 11
    4.6 Discontinuation criteria 11
5. Treatment Plan 11
    5.1 Pretreatment Workup and Staging 11
    5.2 Treatment Protocol 12
    5.3 Adverse Event Monitoring and Follow-up 12
6. Statement of ethics approval 13
7. Statistical analysis 13
8. Monitoring 14
9. Funding and registration 14

Protocol summary

**Study title**	Neoadjuvant Chemoradiotherapy Combined with Surgery vs. Surgery with Adjuvant Therapy for Esophageal Squamous Cell Carcinoma: A Prospective, Randomized Phase III Clinical Trial
**Study phase**	Phase III
**Version number (date)**	V1.0 (September 18th, 2017)
**Institution**	Sichuan Clinical Research Center for Cancer, Sichuan Cancer Hospital & Institute, Sichuan Cancer Center, University of Electronic Science and Technology of China
**Study objectives**	The primary objective of this study is to compare the long-term overall survival (OS) and disease-free survival (DFS) between patients with locally advanced esophageal squamous cell carcinoma (ESCC) who undergo neoadjuvant chemoradiotherapy (NCRT) followed by surgery and those who undergo surgery followed by adjuvant therapy (AT). The secondary objectives include evaluating the treatment-related toxicity, perioperative complications, and pathological outcomes such as pathological complete response (pCR) rates, as well as assessing the impact of pCR on long-term survival.
**Sample size**	206
**Study design**	This is a prospective, randomized, open-label phase III clinical trial aimed at comparing the efficacy and safety of neoadjuvant chemoradiotherapy (NCRT) followed by surgery versus surgery with adjuvant therapy (AT) in patients with locally advanced esophageal squamous cell carcinoma (ESCC). Patients will be randomly assigned to two groups: the NCRT group will receive neoadjuvant chemoradiotherapy (intensity-modulated radiotherapy with concurrent paclitaxel and carboplatin chemotherapy) followed by surgery; the AT group will undergo surgery followed by adjuvant therapy based on pathological staging (chemotherapy or chemoradiotherapy). The primary endpoint of the study is overall survival (OS), and secondary endpoints include disease-free survival (DFS), pathological response, treatment-related toxicity, and perioperative complications. All patients will be followed for at least 5 years after randomization. All study procedures will be conducted in accordance with international clinical trial standards and have been approved by the ethics committee.
**Inclusion criteria**	(1) Histologically confirmed locally advanced resectable thoracic ESCC.<br>(2) Clinical stage cT1N+M0 or T2-4aNxM0 based on the AJCC8-TNM system

	(3) Age between 18 and 75 years<br>(4) At least one measurable lesion in accordance with RECIST 1.1.<br>(5) Eastern Cooperative Oncology Group (ECOG) performance status of 0–1;<br>(6) The expected survival time was > 6 months.<br>(7) No diagnosis of other cancers and no prior anticancer therapy history.<br>(8) There were no operative contraindications.<br>(9) The important organs functions meet the following requirements: the absolute neutrophil count (ANC) ≥ 1.5 × 109/L; the platelet count ≥ 100 × 109/L; hemoglobin ≥ 90 g/L; bilirubin less than or equal to 1.5 times ULN; ALT and AST less than or equal 2.5 times UILN; creatinine clearance rate (CCr) ≥ 50 mL/min; normal thyroid function<br>(10) Female subjects of childbearing potential having a negative pregnancy test result and agreeing to take effective contraceptive measures during the study period and within 3 months after the last dose.<br>(11) Be willing and able to provide written informed consent/assent for the trial.
**Exclusion criteria**	(1) Patients with inoperable tumors or distant metastasis as indicated by preoperative examinations.<br>(2) Patients with enlarged cervical lymph nodes that are biopsy-positive.<br>(3) Patients with a history of other malignancies or who have received other anti-cancer treatments.<br>(4) Patients with infectious diseases requiring treatment.<br>(5) Patients who cannot tolerate surgery based on cardiopulmonary, hepatic, or renal function tests.<br>(6) Patients who have undergone previous gastrectomy and cannot undergo gastric conduit reconstruction.<br>(7) Patients with a history of severe bleeding (blood loss >30 mL within 3 months), hemoptysis (fresh blood >5 mL within 4 weeks), or thromboembolic events (including stroke or transient ischemic attack) within the past 12 months.<br>(8) Patients with a history of or current objective evidence of pulmonary fibrosis, interstitial pneumonia, pneumoconiosis, radiation-induced pneumonia, drug-induced pneumonia, or severe lung function impairment.<br>(9) Patients participating in other clinical trials.<br>(10) Patients with a history of mental illness or substance abuse.<br>(11) Patients who are unable to sign the informed consent form due to psychological, family, or social reasons.

	(12) Other patients who are deemed inappropriate for participation in the study by the investigator.
**Study endpoints**	**Primary Endpoint:**<br><br>Overall Survival (OS): The time from randomization to death from any cause.<br><br>**Secondary Endpoints:**<br><br>Disease-Free Survival (DFS): The time from randomization to disease recurrence or death.<br><br>Pathological Complete Response (pCR): The absence of residual tumor in both the primary tumor site and resected lymph nodes after treatment.<br><br>R0 Resection Rate: The rate of complete resection with no residual tumor at the resection margin.<br><br>Treatment-related Toxicity: The incidence and severity of adverse events, graded according to the Common Terminology Criteria for Adverse Events (CTCAE) version 5.0.<br><br>Perioperative Complications: Including but not limited to anastomotic leakage, pulmonary complications, and surgical site infections.
**Statistical analysis**	All statistical analyses will be conducted using SPSS version 23.0 (IBM Corp) and R version 4.0 (R Foundation for Statistical Computing). A two-sided p-value of less than 0.05 will be considered statistically significant.

1. Study Background

Esophageal cancer, particularly esophageal squamous cell carcinoma (ESCC), is a major cause of cancer-related mortality worldwide. Despite advances in surgical techniques and adjuvant therapies, the prognosis for patients with locally advanced ESCC remains poor, with high rates of recurrence and mortality. Surgical resection remains the cornerstone of treatment, but its efficacy is limited by the high risk of postoperative complications such as anastomotic leakage, and the limited benefit in patients with advanced-stage disease.

Over the past decade, there has been growing interest in the role of neoadjuvant therapy to improve outcomes in patients with locally advanced ESCC. Neoadjuvant chemoradiotherapy (NCRT) has shown promise in downstaging tumors, improving resectability, and reducing the risk of recurrence. This approach aims to treat micrometastatic disease and shrink the tumor before surgery, potentially leading to better outcomes in terms of overall survival (OS) and disease-free survival (DFS).

Adjuvant therapy (AT), including chemotherapy and chemoradiotherapy, has traditionally been used after surgery to address residual disease and improve survival. However, the optimal timing and sequencing of chemotherapy, radiotherapy, and surgery for locally advanced ESCC remain unclear.

A few clinical trials, including the CROSS and NEOCRTEC5010 studies, have demonstrated that NCRT followed by surgery can improve long-term survival. However, few studies have directly compared NCRT followed by surgery with surgery followed by AT in a randomized, prospective setting. The long-term survival outcomes and perioperative complications associated with these two treatment strategies have not been definitively compared in the context of ESCC.

This study seeks to address this gap in knowledge by evaluating and comparing the long-term survival outcomes, treatment-related toxicity, and perioperative complications associated with NCRT followed by surgery versus surgery followed by AT in patients with locally advanced ESCC. Additionally, the study will explore the impact of pathological complete response (pCR) on survival outcomes, which may

provide further insight into the role of NCRT in improving disease control and survival in this patient population..

2. Study Objectives

The primary objective of this study is to compare the long-term overall survival (OS) and disease-free survival (DFS) between patients with locally advanced esophageal squamous cell carcinoma (ESCC) who undergo neoadjuvant chemoradiotherapy (NCRT) followed by surgery and those who undergo surgery followed by adjuvant therapy (AT). The secondary objectives include evaluating the treatment-related toxicity, perioperative complications, and pathological outcomes such as pathological complete response (pCR) rates, as well as assessing the impact of pCR on long-term survival.

2.1 Primary Objective:

Overall Survival (OS): The time from randomization to death from any cause. OS is defined as the time from the patient’s inclusion in the trial until death, regardless of the cause.

2.2 Secondary Objectives:

(1) Disease-Free Survival (DFS): The time from randomization to disease recurrence or death from any cause. DFS is measured as the time from the start of the study until the first event of disease recurrence or metastasis.

(2) Pathological Complete Response (pCR): Defined as the absence of residual tumor cells in the primary tumor site and resected lymph nodes following treatment. pCR is considered a favorable pathological outcome and is associated with improved long-term survival.

(3) R0 Resection Rate: The percentage of patients who achieve complete tumor resection with no residual tumor at the resection margin (pathologically negative margin). An R0 resection indicates that no visible or microscopic tumor remains at the surgical site.

(4) Treatment-Related Toxicity: The incidence and severity of adverse events related to treatment, categorized according to the Common Terminology Criteria for Adverse

Events (CTCAE) version 5.0. This includes hematologic, gastrointestinal, and other toxicities that occur during treatment.

(5) Perioperative Complications: Any complications that arise during the perioperative period, including infections, wound healing issues, and other surgical complications, specifically anastomotic leakage, pulmonary complications, and surgical site infections.

2.3 Exploratory Objectives:

(1) Pathological Downstaging: Defined as the reduction in the size or extent of the tumor, assessed by pathology following neoadjuvant treatment. A significant downstaging is considered indicative of effective treatment.

(2) Biomarker Analysis: The correlation between molecular biomarkers and treatment response or survival outcomes. These biomarkers will be assessed as exploratory endpoints to provide insight into the mechanism of treatment effects.

(3) Health-Related Quality of Life (HRQoL): Assessed using validated questionnaires (e.g., EQ-5D, EORTC QLQ-C30) to measure the physical, emotional, and social well-being of patients throughout the study and after treatment.

3. Study design

This is a prospective, randomized, open-label phase III clinical trial aimed at comparing the efficacy and safety of neoadjuvant chemoradiotherapy (NCRT) followed by surgery versus surgery with adjuvant therapy (AT) in patients with locally advanced esophageal squamous cell carcinoma (ESCC). Patients will be randomly assigned to two groups: the NCRT group will receive neoadjuvant chemoradiotherapy (intensity-modulated radiotherapy with concurrent paclitaxel and carboplatin chemotherapy) followed by surgery; the AT group will undergo surgery followed by adjuvant therapy based on pathological staging (chemotherapy or chemoradiotherapy). The primary endpoint of the study is overall survival (OS), and secondary endpoints include disease-free survival (DFS), pathological response, treatment-related toxicity, and perioperative complications. All patients will be followed for at least 5 years after randomization. All study procedures will be conducted in accordance with international clinical trial standards and have been approved by the ethics committee.

4. Study Population

4.1 Participants

Patients with ESCC who were diagnosed without any prior treatment in Sichuan Cancer Hospital underwent physical examination; gastroscopy; ultrasonic gastroscopy; contrast-enhanced CT scan of the neck, chest, and abdomen; cervical and abdominal ultrasound; esophagography; electrocardiography; and pulmonary function tests. For tumors in the upper esophagus, bronchoscopy was performed to rule out tracheal or bronchial invasion. Fine-needle aspiration of cervical lymph nodes was conducted when metastasis was suspected. If the patient agreed, positron emission tomography-CT(PET/CT) was performed. All patients will be given pretreatment clinical staging according to the 8th UICC for International Cancer Control TNM system. Written informed consent was obtained from all patients before they were recruited.

4.2 Inclusion criteria

(1) Histologically confirmed locally advanced resectable thoracic ESCC.

(2) Clinical stage cT1N+M0 or T2-4aNxM0 based on the AJCC8-TNM system

(3) Age between 18 and 75 years

(4) At least one measurable lesion in accordance with RECIST 1.1.

(5) Eastern Cooperative Oncology Group (ECOG) performance status of 0–1;

(6) The expected survival time was > 6 months.

(7) No diagnosis of other cancers and no prior anticancer therapy history.

(8) There were no operative contraindications.

(9) The important organs functions meet the following requirements: the absolute neutrophil count (ANC) ≥ 1.5 × 109/L; the platelet count ≥ 100 × 109/L; hemoglobin ≥ 90 g/L; bilirubin less than or equal to 1.5 times ULN; ALT and AST less than or equal 2.5 times UILN; creatinine clearance rate (CCr) ≥ 50 mL/min; normal thyroid function

(10) Female subjects of childbearing potential having a negative pregnancy test result

and agreeing to take effective contraceptive measures during the study period and within 3 months after the last dose.

(11) Be willing and able to provide written informed consent/assent for the trial.

4.3 Exclusion criteria

(2) (1) Patients with inoperable tumors or distant metastasis as indicated by preoperative examinations.

(2) Patients with enlarged cervical lymph nodes that are biopsy-positive.

(3) Patients with a history of other malignancies or who have received other anti-cancer treatments.

(4) Patients with infectious diseases requiring treatment.

(5) Patients who cannot tolerate surgery based on cardiopulmonary, hepatic, or renal function tests.

(6) Patients who have undergone previous gastrectomy and cannot undergo gastric conduit reconstruction.

(7) Patients with a history of severe bleeding (blood loss >30 mL within 3 months), hemoptysis (fresh blood >5 mL within 4 weeks), or thromboembolic events (including stroke or transient ischemic attack) within the past 12 months.

(8) Patients with a history of or current objective evidence of pulmonary fibrosis, interstitial pneumonia, pneumoconiosis, radiation-induced pneumonia, drug-induced pneumonia, or severe lung function impairment.

(9) Patients participating in other clinical trials.

(10) Patients with a history of mental illness or substance abuse.

(11) Patients who are unable to sign the informed consent form due to psychological, family, or social reasons.

(12) Other patients who are deemed inappropriate for participation in the study by the investigator.

4.4 Removal criteria

Subjects who are enrolled but violate the protocol should be removed, including:

(1) Mis-diagnosis;

(2) Not meeting the inclusion criteria but meeting the exclusion criteria;

(3) Having incomplete data for evaluation of efficacy and safety.

The reason of rejection should be explained and recorded. However, patients who have received treatment and have safety record should be included in the safety analysis.

4.5 Drop-out/Withdrawal criteria

Subjects could withdraw from the study at any time and for any reason. The investigator could withdraw a subject from the study including the following reasons:

(1) Serious adverse events,

(2) Trial protocol violation,

(3) Poor compliance,

(4) Lack of efficacy,

(5) Trial discontinuation is determined to be necessary for subjects by the investigator.

4.6 Discontinuation criteria

(1) Serious adverse events, or other events that affect subject safety;

(2) Radiographic disease progression or metastasis;

(3) Patients receive other anti-tumor treatments that affect the results of the study;

(4) The subject withdrew his informed consent;

(5) Other conditions that the investigator considers necessary to terminate the study drug treatment.

5. Treatment Plan

5.1 Pretreatment Workup and Staging

Before initiating treatment, all patients underwent comprehensive pretreatment assessment and staging, including physical examination, routine blood tests, esophagogastroduodenoscopy (EGD) with endoscopic ultrasound, contrast-enhanced computed tomography (CT) scans of the neck, chest, and abdomen, cervical ultrasonography, electrocardiography (ECG), echocardiography, and pulmonary function tests. For tumors in the upper esophagus, bronchoscopy was performed to rule out tracheal or bronchial invasion. Fine-needle aspiration of cervical lymph nodes

was conducted when metastasis was suspected. PET-CT was recommended but optional. Clinical staging followed the 8th edition of the UICC TNM classification system 8.

5.2 Treatment Protocol

(1) Neoadjuvant Chemoradiotherapy (NCRT) Group:

Chemotherapy:

Patients in the NCRT group will receive neoadjuvant chemotherapy with paclitaxel (135 mg/m^2) and carboplatin (AUC = 2-5) on Day 1 of each 21-day cycle. This treatment will be administered for two cycles before surgery.

Radiotherapy:

Concurrent with chemotherapy, patients will receive intensity-modulated radiotherapy (IMRT) targeting the primary tumor and involved lymph nodes. The total dose will be 40 Gy delivered in 20 fractions (2 Gy per fraction), five times a week for 4 weeks.

Surgery:

Surgery will be performed 4 to 6 weeks after completing NCRT. The procedure will include McKeown or Ivor Lewis esophagectomy with two-field or three-field lymphadenectomy, depending on the tumor’s extent.

(2) Surgery with Adjuvant Therapy (AT) Group:

Surgery:

Patients in the AT group will undergo McKeown or Ivor Lewis esophagectomy with two-field or three-field lymphadenectomy as per standard surgical procedures.

Adjuvant Therapy:

After surgery, adjuvant therapy will be based on the pathological staging of the tumor. Adjuvant therapy may include chemotherapy or chemoradiotherapy, following the NCCN guidelines (Version 4.2017).

5.3 Adverse Event Monitoring and Follow-up

During neoadjuvant therapy, adverse events (AEs) will be monitored monthly according to the Common Terminology Criteria for Adverse Events (CTCAE) version 4.0. Postoperative complications occurring within 30 days will be assessed using the

Clavien-Dindo classification system. Follow-up for recurrence or death will be conducted every three months for the first two years and every six months from the third to fifth years.

6. Statement of ethics approval

This study was approved by the ethics committee of Sichuan Cancer Hospital, China. The ethics certificate number is SCCHEC-KY-2017-043. All the included patients signed informed consent forms.

7. Statistical analysis

(1) Sample Size Calculation

As one of the participating centers in the NEOCRTEC5010 trial, we conducted a sample size calculation based on internal data and the JCOG9907 trial. We assumed a 5-year OS rate of 60% for patients assigned to the NCRT group and 43% for those assigned to the AT group. With a one-sided type I error of 0.05 and a power of 80%, using a randomization ratio of 1:1 between the experimental and control arms, and accounting for a 5% dropout rate, the intended number of randomly assigned patients was 206 (103 per arm). The calculations were performed assuming an exponential distribution.The sample size calculation assumed an exponential distribution for survival times.

(2) Statistical Analysis

OS and DFS were calculated using the Kaplan-Meier method and compared using the log-rank test. Categorical variables between groups were compared using the X^2 test or Fisher's exact test, while continuous variables were assessed using t-tests or analysis of variance. A P-value of < 0.05 was considered statistically significant. The Cox proportional hazards model was employed to calculate hazard ratios (HRs) and their 95% confidence intervals (CIs). Statistical analyses were performed using SPSS version 26.0 (IBM Corp) and R version 4.2.3.

8. Monitoring

The Good Clinical Practice (GCP) board and ethics committee of Sichuan Cancer Hospital evaluated patient safety, trial progress, and data integrity. The GCP reviewed the trial data every three months. The principal investigators (PI) were responsible for the design and performance of the study.

9. Funding and registration

This study was supported by Science and Technology Department of Sichuan Province (Grant No. 2018SZ0199).

This trial has been registered and released in ClinicalTrials.gov (Identifier NCT06775652).
