# Supplementary material for: Long-Term Survival Outcomes of NCRT With Surgery vs Surgery With Adjuvant Therapy for ESCC: A Single-Center Prospective Phase 3 Randomized Clinical Trial
Source: JAMA Netw Open. 2026 Jan 5;9(1):e2550307. doi: 10.1001/jamanetworkopen.2025.50307 (PMC12771256; doi:10.1001/jamanetworkopen.2025.50307)
Supplement: Supplement 2. — eTable 1. Patient Characteristics eTable 2. Adverse Events Between NCRT and AT Groups eTable 3. Surgical and Pathological Findings eTable 4. Postoperative Complications [file jamanetwopen-e2550307-s002.pdf]

Supplemental Online Content

He W, Li Z, Xie Q, et al. Long-term survival outcomes of NCRT with surgery vs surgery with adjuvant therapy for ESCC: a single-center prospective phase 3 randomized clinical trial. *JAMA Netw Open.* 2025;8(12):e2550307. doi:10.1001/jamanetworkopen.2025.50307

eTable 1. Patient Characteristics

eTable 2. Adverse Events Between NCRT and AT Groups

eTable 3. Surgical and Pathological Findings

eTable 4. Postoperative Complications

This supplemental material has been provided by the authors to give readers additional information about their work.

© 2025 He W et al. *JAMA Network Open*.

eTable 1. Patient Characteristics

		NCRT (n=118)	AT (n=112)
Sex	Male	102 (86.4)	97 (86.6)
	Female	16 (13.6)	15 (13.4)
Age (IQR)		62 (54-66)	63 (55-66)
Site	Upper	15(12.7)	7 (6.3)
	Middle	71 (60.2)	55 (49.1)
	Lower	32 (27.1)	50 (44.6)
cT stage	T1	4 (3.4)	6 (5.4)
	T2	16 (13.6)	22 (19.6)
	T3	85 (72.0)	75 (67.0)
	T4	13 (11.0)	9 (8.0)
cN stage	N0	14 (11.9)	25 (22.3)
	N1	60 (50.8)	43 (38.4)
	N2	44 (37.3)	43 (38.4)
	N3	0 (0.0)	1 (0.9)
cTNM stage	I	3 (2.6)	3 (2.7)
	II	28 (23.7)	37 (33.0)
	III	74 (62.7)	63 (56.3)
	IVA	13 (11.0)	9 (8.0)
Neoadjuvant chemotherapy cycles	1	8 (6.8)	NaN
	2	101 (85.6)	NaN

© 2025 He W et al. *JAMA Network Open*.

	3	3 (2.5)	NaN
	4	6 (5.1)	NaN
Adjuvant treatment modality	None	NaN	9 (8.0)
	Chemotherapy	NaN	23 (20.5)
	Radiotherapy	NaN	15 (13.4)
	Chemoradiotherapy	NaN	65 (58.1)
Adjuvant chemotherapy cycles	1	NaN	17 (19.3)
	2	NaN	43 (48.9)
	3	NaN	12 (13.6)
	4	NaN	12 (13.6)
	5	NaN	2 (2.3)
	6	NaN	2 (2.3)

Data are presented as n (%) or IQR interquartile range; BMI body mass index; NCRT neoadjuvant chemoradiotherapy; AT adjuvant therapy.

© 2025 He W et al. *JAMA Network Open*.

eTable 2. Adverse Events Between NCRT and AT Groups

		NACT (n=118)	AT (n=103)	*p*
Anemia	None	47 (39.8)	49 (47.6)	0.51
	Grade 1-2	68 (57.6)	52 (50.5)	
	Grade 3-4	3 (2.6)	2 (1.9)	
Leukopenia	None	16 (13.6)	22 (21.4)	0.17
	Grade 1-2	60 (50.8)	54 (52.4)	
	Grade 3-4	42 (35.6)	27 (26.2)	
Neutropenia	None	37 (31.4)	45 (43.7)	0.14
	Grade 1-2	43 (36.4)	34 (33.0)	
	Grade 3-4	38 (32.2)	24 (23.3)	
Thrombocytopenia	None	88 (74.6)	77 (74.8)	0.60
	Grade 1-2	25 (21.2)	24 (23.3)	
	Grade 3-4	5 (4.2)	2 (1.9)	
Hepatic dysfunction	None	87 (73.7)	77 (74.8)	0.98
	Grade 1-2	31 (26.3)	26 (25.2)	
	Grade 3-4	0(0)	0(0)	
Anorexia	None	55 (46.6)	68 (66.0)	0.01
	Grade 1-2	59 (50.0)	34 (33.0)	
	Grade 3-4	4 (3.4)	1 (1.0)	
Vomiting	None	92 (78.0)	93 (90.3)	0.04
	Grade 1-2	25 (21.2)	10 (9.7)	
	Grade 3-4	1 (0.8)	0 (0.0)	
Diarrhea	None	111 (94.1)	95 (92.2)	0.79

© 2025 He W et al. *JAMA Network Open*.

	Grade 1-2	7 (5.9)	8 (7.8)	
	Grade 3-4	0(0)	0(0)	
Constipation	None	105 (89.0)	92 (89.3)	1.00
	Grade 1-2	13 (11.0)	11 (10.7)	
	Grade 3-4	0(0)	0(0)	
Fatigue	None	58 (49.2)	67 (65.0)	0.05
	Grade 1-2	57 (48.3)	35 (34.0)	
	Grade 3-4	3 (2.5)	1 (1.0)	
Radiation esophagitis	None	72 (61.0)	53 (66.3)	0.38
	Grade 1-2	38 (32.2)	25 (31.2)	
	Grade 3-4	8 (6.8)	2 (2.5)	

Data are presented as n (%); NCRT neoadjuvant chemoradiotherapy; AT adjuvant therapy. Adverse events were graded according to the National Cancer Institute's Common Terminology Criteria for Adverse Events, version 3.0.

© 2025 He W et al. *JAMA Network Open*.

eTable 3. Surgical and Pathological Findings

		NACT (n=118）	AT (n=112）	*p*
Approach	MIE	106 (89.8)	100 (89.3)	1.00
	Thoracotomy	12 (10.2)	12 (10.7)	
Esophagectomy	McKeown	110 (93.2)	101 (90.2)	0.55
	IvorLewis	8 (6.8)	11 (9.8)	
RLNs (IQR)		16 (11-22)	20 (13-29)	<0.001
p/ypT stage	T0	42 (35.6)	0 (0.0)	<0.001
	T1	20 (16.9)	8 (7.1)	
	T2	18 (15.3)	23 (20.5)	
	T3	36 (30.5)	78 (69.7)	
	T4a	2 (1.7)	3 (2.7)	
p/ypN stage	N0	84 (71.2)	31 (27.7)	<0.001
	N1	24 (20.3)	55 (49.1)	
	N2	9 (7.6)	18 (16.1)	
	N3	1 (0.9)	8 (7.1)	
p/ypTNM stage	I	64 (54.2)	1 (0.9)	<0.001
	II	19 (16.1)	37 (33.0)	
	III	33 (28.0)	66 (58.9)	
	IV	2 (1.7)	8 (7.2)	
Vascular cancer thrombus	No	108 (91.5)	63 (56.2)	<0.001
	Yes	10 (8.5)	48 (42.9)	
	unknown	0 (0.0)	1 (0.9)	
Neural invasion	No	96 (81.4)	67 (59.8)	0.001

© 2025 He W et al. *JAMA Network Open*.

	Yes	22 (18.6)	44 (39.3)	
	unknown	0 (0.0)	1 (0.9)	
Resection margin	R0	116 (98.3)	108 (96.4)	0.63
	R1/R2	2 (1.7)	4 (3.6)	
pCR	No	84 (71.2)	NaN	NaN
	Yes	34 (28.8)	NaN	

Data are presented as n (%) or IQR interquartile range; MIE minimally invasive esophagectomy; RLNs removed lymph nodes.

Data are presented as n (%) or IQR interquartile range; NCRT neoadjuvant chemoradiotherapy; AT adjuvant therapy; pCR Pathological complete response.

© 2025 He W et al. *JAMA Network Open*.

eTable 4. Postoperative Complications

	NACT (n=118)	AT (n=112)	*p*
Anastomotic leakage			
No	105 (89.0)	96 (85.7)	0.58
Yes	13 (11.0)	16 (14.3)	
Recurrent laryngeal nerve injury			
No	112 (94.9)	109 (97.3)	0.55
Yes	6 (5.1)	3 (2.7)	
Chylothorax			
No	113 (95.8)	108 (96.4)	1.00
Yes	5 (4.2)	4 (3.6)	
Lung infection			
No	97 (82.2)	94 (83.9)	0.86
Yes	21 (17.8)	18 (16.1)	
Pleural effusion			
No	95 (80.5)	91 (81.2)	1.00
Yes	23 (19.5)	21 (18.8)	
Arrhythmia			
No	108 (91.5)	105 (93.8)	0.70
Yes	10 (8.5)	7 (6.2)	
Pneumothorax			
No	116 (98.3)	111 (99.1)	1.00
Yes	2 (1.7)	1 (0.9)	

Data are presented as n (%); NCRT neoadjuvant chemoradiotherapy; AT adjuvant therapy.

© 2025 He W et al. *JAMA Network Open*.
