# Supplementary material for: Long-Term Survival Outcomes of NCRT With Surgery vs Surgery With Adjuvant Therapy for ESCC: A Single-Center Prospective Phase 3 Randomized Clinical Trial
Source: JAMA Netw Open. 2026 Jan 5;9(1):e2550307. doi: 10.1001/jamanetworkopen.2025.50307 (PMC12771256; doi:10.1001/jamanetworkopen.2025.50307)
Supplement: Supplement 3. — Data Sharing Statement [file jamanetwopen-e2550307-s003.pdf]

Data Sharing Statement

He. Long-Term Survival Outcomes of NCRT With Surgery vs Surgery With Adjuvant Therapy for ESCC. *JAMA Netw Open*. Published December 18, 2025.
doi:10.1001/jamanetworkopen.2025.50307

Data
Additional Information: ClinicalTrials.gov Identifier: NCT06775652.
Data available: Yes
Data types: Deidentified participant data
How to access data: Deidentified individual participant data, along with the study protocol and statistical analysis plan, will be made available to qualified researchers whose proposed use of the data has been approved by the corresponding author and the institutional review board. Data will be shared beginning 6 months after publication and ending 2 years thereafter, for academic purposes only. Requests should be directed to the corresponding author.
When available: With publication

Supporting Documents
Document types: None

Additional Information
Who can access the data: Deidentified individual participant data, will be made available to qualified researchers whose proposed use of the data has been approved by the corresponding author and the institutional review board.
Types of analyses: For academic purposes only
Mechanisms of data availability: After approval of a proposal, or with a signed data access agreement.
